# Rate or Rhythm Control in CRT (RHYTHMIC): Study rationale and protocol

**DOI:** 10.1016/j.hroo.2022.09.001

**Published:** 2022-09-13

**Authors:** Mark K. Elliott, Felicity de Vere, Vishal S. Mehta, Nadeev Wijesuriya, Marina Strocchi, Ronak Rajani, Steven Niederer, Christopher A. Rinaldi

**Affiliations:** ∗ School of Biomedical Engineering and Imaging Sciences, King’s College London, London, United Kingdom; † Department of Cardiology, Guy’s and St Thomas’ NHS Foundation Trust, London, United Kingdom

**Keywords:** Cardiac resynchronization therapy, Atrial fibrillation, Biventricular pacing percentage, Atrioventricular synchrony, Atrial fibrillation ablation, Atrioventricular node ablation

## Abstract

**Background:**

Atrial fibrillation (AF) has several detrimental effects on heart failure patients treated with cardiac resynchronization therapy (CRT). These include suboptimal biventricular pacing and the loss of atrioventricular (AV) synchrony. AV node ablation improves biventricular pacing and clinical outcomes in large observational studies. However, restoration of sinus rhythm with AF ablation may have additional benefits.

**Objectives:**

To compare the effects of AV node ablation and AF ablation on echocardiographic and symptomatic outcomes in patients with CRT and suboptimal biventricular pacing.

**Methods:**

RHYTHMIC is a multicenter prospective randomized controlled trial. Seventy patients will be recruited and randomized to each ablation strategy in a 1:1 ratio. Key inclusion criteria include a previous CRT implant (with atrial lead) for dyssynchronous heart failure, and biventricular pacing <95% secondary to AF. Patients with permanent AF will be excluded.

**Results:**

Patients will undergo baseline assessment including transthoracic echocardiography (TTE), device check, blood tests, electrocardiogram (ECG), 6-minute walk test, and symptom questionnaire. They will then undergo either AV node ablation or AF ablation according to their allocated group. Follow-up will occur at 1 week (TTE and ECG) and at 6 months (repeat of baseline investigations). The primary endpoint will be change in left ventricular ejection fraction on TTE.

**Conclusion:**

This is the first randomized controlled trial comparing AV node ablation and AF ablation in patients with CRT. We anticipate it will provide valuable insight into the management of this frequently encountered clinical scenario in a challenging patient cohort.

**Key Findings**

▪ Atrial fibrillation (AF) is associated with poorer response and increased mortality after cardiac resynchronization therapy (CRT) owing to a reduction in biventricular pacing and loss of atrioventricular (AV) synchrony.
▪ There is evidence supporting the use of AV node ablation in patients with AF and heart failure treated with CRT.
▪ There is also early evidence from the general heart failure population suggesting a benefit for AF ablation; however, the optimal treatment strategy is unclear.
▪ The RHYTHMIC study is a randomized controlled trial that will directly compare AF ablation and AV node ablation for heart failure patients with CRT and suboptimal biventricular pacing.

## Introduction

Patients with atrial fibrillation (AF) were largely excluded from the landmark trials of cardiac resynchronization therapy (CRT).^1^ However, up to a third of patients receiving CRT in clinical practice have AF.^2^ Heart failure and AF have a complex synergistic interaction, with each influencing the progression of the other. Elevated left atrial pressures caused by left ventricular (LV) dysfunction induce structural and electrical remodeling, which create the substrate for AF. In turn, AF causes rapid and irregular LV activation and the loss of atrial systole, both of which can reduce cardiac output.^1^ The prevalence of AF increases with heart failure severity^3^ and is associated with poorer outcomes, both in the general heart failure population^4^ and in those with electrical dyssynchrony who receive CRT.^5^^,^^6^ Randomized evidence for the use of CRT in patients with AF is limited. In a substudy of the Resynchronization/Defibrillation for Ambulatory Heart Failure Trial (RAFT), 229 patients with permanent AF and dyssynchronous heart failure were randomized to either CRT-defibrillator or implantable cardioverter-defibrillator (ICD) alone.^7^ There was no significant difference in mortality between the groups, and only a borderline significant trend toward fewer heart failure hospitalizations for those who received CRT. However, it should be noted that only 1 patient received an atrioventricular (AV) node ablation, and satisfactory biventricular pacing (>95%) was achieved in only a third of patients. In a recent meta-analysis of more than 80,000 patients, the presence of AF at implant was associated with a significantly higher mortality rate after CRT.^5^ Current guidelines have a class IIa recommendation for CRT in patients with AF and LV ejection fraction ≤35% who meet CRT criteria, provided a strategy is in place to ensure biventricular capture.^8^^,^^9^

AF has a variety of deleterious effects on the delivery of CRT. Firstly, rapid and irregular activation of the ventricles can reduce the delivery of biventricular pacing. In a large study of more than 30,000 patients, atrial arrhythmias were the most common cause for having a biventricular pacing percentage of less than 95%.^10^ Registry studies have consistently shown that suboptimal biventricular pacing is associated with higher mortality rates after CRT, with even very small decreases in CRT delivery affecting outcomes. Low biventricular pacing percentage has also been associated with lower rates of LV reverse remodeling.^11^ In an observational study of 36,935 CRT patients followed up for a mean of 587 ± 324 days, those with biventricular pacing <95% had a 19% increase in mortality (hazard ratio [HR] 1.19; *P* < .001).^12^ Interestingly, patients with AF who had seemingly high biventricular pacing delivery (>98.5%) had a higher mortality rate than those in sinus rhythm with equivalent pacing percentage. This may be related to the fact that biventricular pacing percentage provided on standard device counters likely underestimates true CRT delivery, owing to the presence of fusion and pseudo-fusion beats.^13^ This highlights that the problem of suboptimal CRT delivery in patients with AF is likely underestimated in clinical practice. Secondly, the loss of atrial systole in patients with AF may also attenuate the benefits of CRT. Atrial systole is thought to contribute up to 20%–30% of cardiac output.^14^ Small observational studies have demonstrated the acute hemodynamic benefits of optimizing AV delays during CRT,^15^, ^16^, ^17^ and a recent mechanistic study of 19 patients undergoing His bundle pacing suggested that the majority of the hemodynamic benefit provided by CRT was attributable to shortening of the AV delay, rather than ventricular resynchronization.^18^ It is therefore likely that patients with persistent or permanent AF fail to benefit from this important aspect of CRT. Thirdly, for patients who receive a CRT-defibrillator, AF is an important cause of inappropriate ICD shocks.^19^, ^20^, ^21^ These can have a significant impact on patient quality of life and burden on healthcare services, and have been independently associated with increased long-term mortality.^19^, ^20^, ^21^

As for any patient with AF, the management strategy for those with CRT is either rate or rhythm control. For patients who fail to achieve adequate control with AV blocking agents such as beta blockers or digoxin, invasive management with an AV node ablation completely eliminates AV conduction and renders the patients dependent on pacing. Although this can often achieve biventricular pacing of close to 100%, the presence of ventricular ectopy or tachycardias can still result in suboptimal CRT delivery. There is strong evidence from large observational studies that AV node ablation is associated with reduced mortality after CRT. The multicenter CERTIFY study^22^ examined mortality outcomes after CRT in 7384 patients, and compared those with AF who were treated medically, those with AF who had an AV node ablation, and those in sinus rhythm. Patients with AF who underwent an AV node ablation had significantly lower mortality compared to those treated medically, and outcomes were comparable to those in sinus rhythm. Although no randomized studies have been performed, these findings are supported by a large meta-analysis of more than 80,000 patients^5^ and are reflected in current European guidelines, where AV node ablation has a class IIa indication in case of incomplete biventricular pacing after CRT.^8^ More recently, the randomized APAF-CRT trial^23^ demonstrated a significant reduction in all-cause mortality with AV node ablation and CRT implantation vs pharmacological rate control for patients with severely symptomatic permanent AF and narrow QRS (<110 ms) and at least 1 heart failure hospitalization in the previous year. Although this is a different patient cohort from those with dyssynchronous heart failure and existing CRT devices, it provides further evidence for the benefits of this ablation approach. AV node ablation has also been shown to significantly reduce the risk of both inappropriate and appropriate shocks in patients with CRT-defibrillators.^24^

Rhythm control may have advantages over rate control for patients with CRT, as atrial systole can be restored. Although rhythm control can be achieved with electrical cardioversion and antiarrhythmic therapy, many antiarrhythmic drugs are contraindicated in patients with LV impairment, and long-term maintenance of sinus rhythm likely requires left atrial ablation. AF ablation is feasible in patients with CRT. In a small observational study by Fink and colleagues,^25^ 38 nonresponders to CRT who had AF underwent pulmonary vein isolation. Significant improvements were observed in biventricular pacing percentage, LV ejection fraction, and New York Heart Association (NYHA) class, and 67% of patients were free from AF at 24 months. Randomized studies of AF ablation in the CRT population are lacking; however, evidence can be inferred from trials in the general heart failure population. In the PABA-CHF study,^26^ 81 patients with AF and LV impairment (ejection fraction <40%) were randomized to either AF ablation or AV node ablation with CRT implantation. Patients in the AF ablation group were found to have superior improvements in LV ejection fraction, 6-minute walk test, and heart failure symptoms. In the CASTLE-AF study,^27^ 398 patients with symptomatic paroxysmal or persistent AF and severe LV impairment were randomized to AF ablation or medical therapy. There was a significant reduction in the primary composite endpoint of death or heart failure hospitalization in the ablation group (HR 0.62; *P* = .007), and 63% of patients were free from AF on device interrogation after 60 months of follow-up. 27.5% of patients in this study had a CRT device, and there was a nonsignificant trend toward a lower primary endpoint in this subgroup (HR 0.54; 95% confidence interval 0.28–1.04). However, questions have been raised about the real-world applicability of the findings, given that only 13% of patients screened for the study met inclusion criteria. No study has yet compared rhythm vs rate control strategies in patients with AF and CRT. The RHYTHMIC study seeks to compare AV node ablation with AF ablation for the management of patients with CRT and suboptimal biventricular pacing secondary to AF. We hypothesize that the restoration of AV synchrony with AF ablation will lead to superior LV reverse remodeling compared to AV node ablation.

## Objectives

The primary research objective is to compare the effects of AV node ablation and AF ablation on echocardiographic and symptomatic outcomes in patients with CRT and suboptimal (<95%) biventricular pacing. The principal hypothesis tested will be that AF ablation is superior to AV node ablation in improving LV ejection fraction (primary endpoint). Secondary endpoints will include changes in LV ejection fraction and LV end-systolic volume at 1 week; and changes in LV end-systolic volume, Minnesota Living with Heart Failure Questionnaire Score, NYHA class, 6-minute walk test, NT-proBNP, AF burden, biventricular pacing percentage, ventricular arrhythmias, and inappropriate ICD shocks at 6 months. Procedural complications will be recorded, and exploratory safety outcome differences will be analyzed.

If there is no overall benefit for AF ablation on intention-to-treat analysis, it is important to determine if this is because restoring sinus rhythm has no benefit over rate control, or if the benefits of AF ablation are diluted by a high rate of recurrence. We will therefore perform a subanalysis comparing the clinical endpoints between patients who underwent AF ablation and are in sinus rhythm at 6 months, vs those who underwent AF ablation and are in AF at 6 months, vs those who underwent AV node ablation. If significant benefits are observed for patients who maintain sinus rhythm after AF ablation, the next research objective will be to determine which baseline factors are most useful to predict the chance of success after AF ablation in this patient cohort. Baseline factors tested will include clinical factors (eg, duration of AF, paroxysmal vs persistent AF, age, comorbidities) and imaging characteristics on echocardiography (eg, LV size, LV function, left atrial size). We will also assess if improvement in LV ejection fraction correlates with a reduction in AF burden.

## Design

The RHYTHMIC study is a multicenter, prospective randomized controlled trial comparing AV node ablation and AF ablation in patients with CRT and suboptimal (<95%) biventricular pacing secondary to AF. The study conforms to the Declaration of Helsinki, and ethical approval has been granted by the Health Research Authorities and London – Chelsea Research Ethics Committee (21/LO/0116). The study is funded by the European Research Council (864055) and British Heart Foundation (RG/20/4/34803).

### Trial design

Seventy patients with existing CRT devices (implanted for dyssynchronous heart failure) and suboptimal (<95%) biventricular pacing due to AF will be included in the study. Patients will be randomized to either AV node ablation or AF ablation in a 1:1 ratio and will be followed up at 6 months.

### Eligibility

**Figure 1:**
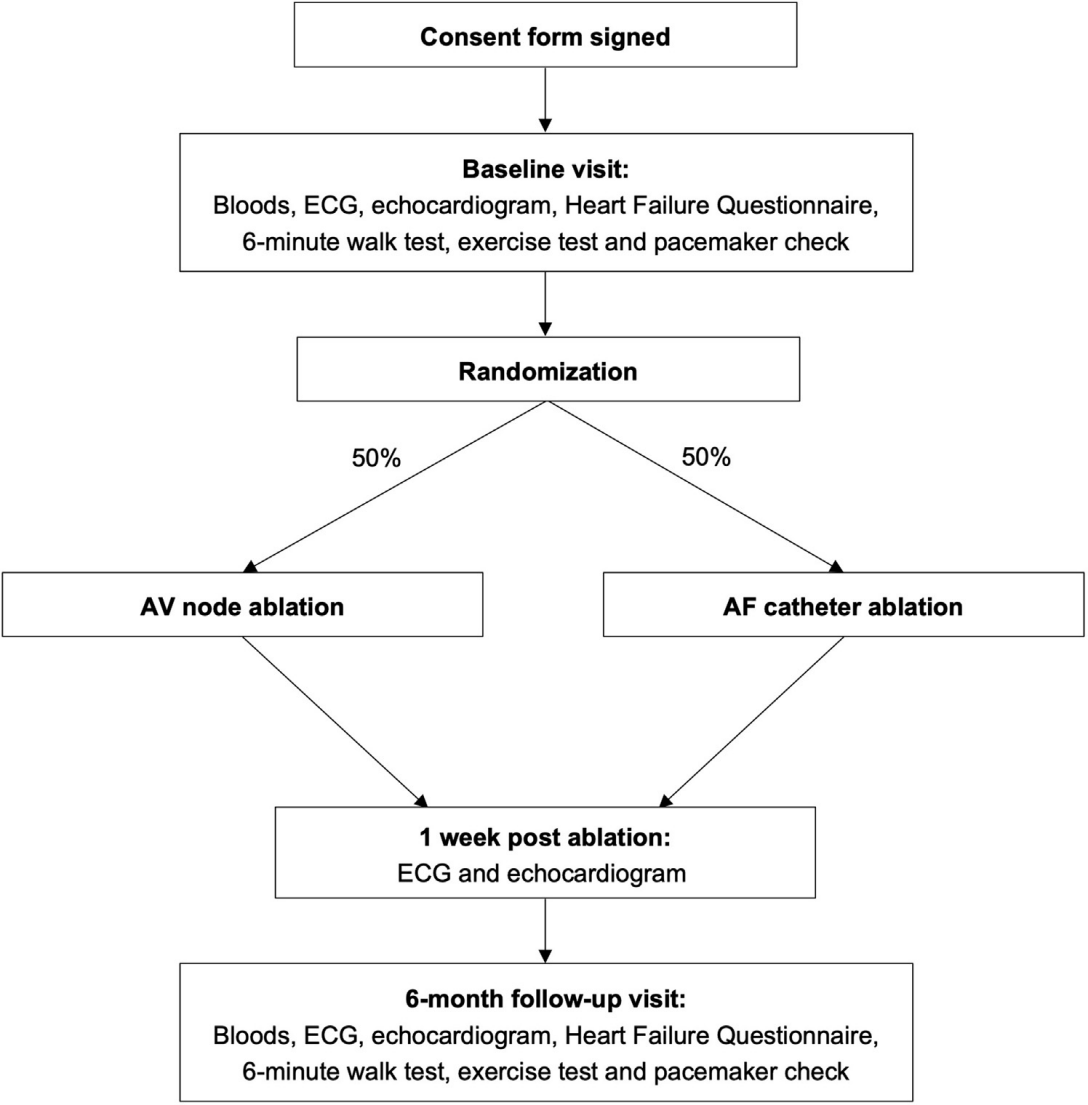
Flow diagram of study protocol. AF = atrial fibrillation; AV = atrioventricular; ECG = electrocardiogram.

Inclusion and exclusion criteria are shown in Table 1. Eligible patients identified via cardiology outpatient clinics, pacing clinics, or inpatient wards will be informed about the study and provided with a patient information sheet. After reading the information sheet and after questions have been addressed, eligible patients will provide written informed consent for participation in the study. The protocol and data collection are summarized in Figure 1. Both conventional CRT “responders” and “nonresponders” will be included, as patients may experience a deterioration in biventricular pacing owing to new-onset or worsening AF after the initial assessment of response.

**Table 1:** Patient eligibility

Inclusion criteria	Exclusion criteria
Age 18–85 years	Life expectancy <1 year
Ability to provide informed consent to participate and willing to comply with the clinical investigation plan and follow-up schedule	Permanent atrial fibrillation
QRS duration >120 ms on surface ECG, severe left ventricular systolic impairment (EF ≤35%), and clinical symptoms of heart failure despite optimum medical therapy (NYHA class II–IV) at time of CRT implant or upgrade	Presence of atrial or ventricular thrombus
Successful CRT implant or upgrade including atrial lead	Mechanical aortic valve replacement
Biventricular pacing percentage <95% secondary to atrial fibrillation at least 3 months post implant or upgrade, despite optimal pharmacological rate control	Severe peripheral vascular disease
Clinically indicated for AV node ablation	Female participants who are pregnant, lactating, or planning pregnancy during the course of the study
	LA diameter >6 cm
	Persistent AF duration >3 years
	Participation in other studies with active treatment / investigational arm

AF = atrial fibrillation; AV atrioventricular; CRT = cardiac resynchronization therapy; ECG = electrocardiogram; EF = ejection fraction; LA = left atrial; NYHA = New York Heart Association.

### Baseline data collection

At baseline visit the patients will undergo clinical assessment, medication history, electrocardiogram (ECG), 6-minute walk test, Minnesota Living with Heart Failure Questionnaire, blood tests (including serum NT-proBNP), device check, and transthoracic echocardiogram. Cardiac device metrics collected will include AF burden, AF duration, biventricular pacing percentage, and previous ICD therapies. Echocardiographic metrics will include 2-dimensional LV end-diastolic volume, LV end-systolic volume, LV ejection fraction, and left atrial size. After the baseline visit, patients will be randomized 1:1 using dedicated software to either AV node ablation or AF ablation. Randomization will be stratified using the minimization method by heart failure etiology (ischemic or nonischemic) and by AF type (paroxysmal or persistent).

### Ablation procedure

Patients will receive either AV node ablation or AF ablation according to randomization. For patients undergoing AF ablation, either cryoablation or radiofrequency ablation is permitted. They will undergo isolation of all 4 pulmonary veins. Additional lesions considered indicated by the operator are permitted. Patients in AF at the end of the procedure may undergo DC cardioversion to achieve sinus rhythm. Programming of AV delays after restoration of sinus rhythm will be left to the discretion of the operator.

### Follow-up

Patients will undergo transthoracic echocardiogram and ECG 1 week post ablation, to assess acute response. They will then be seen at the 6-month visit, where they will undergo repeat clinical assessment, ECG, 6-minute walk test, blood tests (including serum NT-proBNP), device check, and transthoracic echocardiogram. Patients in the AF ablation arm who have a recurrence of AF during the follow-up period will be permitted 1 re-do ablation, and their follow-up visit will occur 6 months after the second procedure.

### Sample size justification

Previous comparisons between AV node ablation and AF ablation in patients with AF and heart failure (not indicated for CRT) found a -1% change in LV ejection fraction with AV node ablation compared to 8% with AF ablation.^26^ Measurements of changes in ejection fraction in CRT patients receiving AV node ablation or AF ablation have a variability of 7.1%–10.7%,^25^^,^^28^ giving a maximum variability of 11%. Assuming a normal distribution, a group size of 32 for each arm would achieve 90% power to reject the null hypothesis of equal means, with an alpha value of 0.05 to detect the 9% difference in change in LV ejection fraction using a 2-sided independent *t* test. This sample size will also ensure satisfactory 80% statistical power for detection of 7.5% difference in change in LV ejection fraction. To allow for an approximately 10% drop-out rate we will recruit a total of 70 patients.

### Statistical analysis

Changes in all cardiac metrics between arms will be compared using *t* tests for independent samples. An intention-to-treat analysis will be performed for the primary and secondary endpoints. Subgroup analyses will be performed for important covariates, including heart failure etiology, sex, age, paroxysmal vs persistent AF, and duration of AF. Subsequently, prediction of outcomes will be performed using regression analyses, considering potential confounders and/or mediators. This procedure will have 2 steps. First, we will conduct exploratory analyses through the use of univariate models and graphical checks of pattern between each candidate predictor and outcome variable. On the basis of these results, and after checking that linearity assumptions are correct, we will examine multivariable associations, taking account of potential confounding factors. Predictors in univariate analysis with *P* < .10 will be further tested in a prespecified multivariable regression model. Predictors will be entered into the model in groups determined by time ordering and/or their clinical relevancy. *P* < .05 will be considered statistically significant.

## Discussion

At present there are no randomized studies comparing AV node ablation and AF ablation in patients with CRT and AF. RHYTHMIC is therefore an important trial that will help address a frequently encountered clinical scenario. Large observational trials support the use of AV node ablation in patients with CRT and AF,^5^^,^^22^ and this is reflected in current clinical guidance.^29^ The role for AF ablation in patients with heart failure is increasingly recognized, though its use in patients with CRT has not been widely studied. Although both AV node ablation and AF ablation are likely to increase biventricular pacing percentage, AF ablation may have the additional benefit of restoring AV synchrony. If the RHYTHMIC study demonstrates clinical superiority for AF ablation over AV node ablation, this will be an important hypothesis-generating result that will justify larger randomized studies with robust morbidity and mortality endpoints.

If the trial shows no significant difference between the 2 ablation strategies, it will be important to determine if this is because there is no additional benefit for AF ablation over AV node ablation, or if the clinical benefit is diminished by a high rate of AF recurrence. Success rates after AF ablation in randomized studies of heart failure patients vary from 50% to 88%.^30^ Furthermore, an international multicenter registry demonstrated that the presence of heart failure reduces success rates after AF ablation in patients with persistent AF (57.3% vs 75.8%), though the equivalent effect for patients with paroxysmal AF was marginal and not statistically significant (78.7% vs 85.7%).^31^ If patients in the AF ablation arm who maintain sinus rhythm at 6-month follow-up display superior echocardiographic remodeling and symptomatic response compared to the AV node ablation group, this will suggest a potential benefit for AF ablation in a select group of patients who have a low risk of AF recurrence after ablation. This would justify further study with a randomized trial focused on this select patient cohort. A variety of factors are known to affect the risk of recurrence after AF ablation in patients with heart failure, including patient age, duration of AF, comorbidities, and structural and electrical left atrial remodeling.^30^ Subanalyses of the RHYTHMIC study provide additional information on which factors predict outcome after AF ablation in patients with CRT devices. We recognize that patient numbers may limit the statistical power to predict outcomes; however, this study may still provide useful hypothesis-generating data to guide the design of larger trials.

The definition of “success” for AF ablation varies between studies and is often related to recurrence of a >30-second episode of atrial arrhythmia during the follow-up period.^32^ Given that all enrolled patients have CRT devices in situ, the RHYTHMIC study has the advantage of allowing continuous rhythm monitoring after ablation, and therefore the effect of ablation on AF burden can be determined. In a recent study of 207 patients with AF and existing pacemakers, ICDs, or implantable loop recorders, AF ablation significantly reduced the burden of AF in patients with both paroxysmal and persistent AF at 1-year follow-up (median AF burden 1.05% vs 0.10% and 99.9% vs 0.30%, respectively).^33^ Significant reductions compared to pre ablation were also found at 4-year follow-up (0.10% and 87.3%, respectively). In the context of patients with heart failure, persistent AF, and CRT, although long-term sinus rhythm maintenance may be difficult to achieve, a reduction in AF burden may be sufficient to achieve a benefit owing to improved biventricular pacing and atrioventricular synchrony. To this end, we will examine if changes in AF burden correlate with improvements in LV ejection fraction.

Any additional clinical benefit for AF ablation over AV node ablation will need to be carefully balanced against the risk of complications, recurrence rate, and need for repeat procedures. AF ablation is a longer and more invasive procedure than AV node ablation, requiring transseptal puncture and, often, transesophageal echocardiography and general anesthesia. AF ablation carries a 2%–3% risk of potentially life-threatening complications,^34^^,^^35^ while complication rates for AV node ablation are comparatively lower.^36^ In the CASTLE-AF study, repeat ablations were performed in 24.5% of patients assigned to AF ablation during the study period, with an overall recurrence rate of 50% at 60-month follow-up.^27^ It is therefore likely that a significant proportion of heart failure patients with CRT who undergo AF ablation will require multiple procedures, and may ultimately require AV node ablation owing to AF recurrence. To justify the increased risk to the patient, along with increased time and financial burden on healthcare systems, the clinical benefits for AF ablation would need to be clear and substantial. Although the cost-effectiveness of AF ablation has been debated, a recent analysis estimated a favorable incremental cost-effectiveness ratio for patients with AF and heart failure of £6438 per quality-adjusted life year within the UK National Health Service.^37^ The cost-effectiveness of AV node ablation for patients with existing CRT devices is not well studied, and results data from the RHYTHMIC study will allow assessment of this important factor, along with comparison with AF ablation.

## Conclusion

The RHYTHMIC study is a multicenter, prospective randomized controlled trial comparing AV node ablation and AF ablation for patients with heart failure and CRT who have low biventricular pacing. This is the first randomized trial to compare these 2 commonly used treatment strategies, and will help answer an important and frequently encountered clinical question. The results of the study will aid the future design of larger trials with hard morbidity and mortality endpoints, and help shape clinical decision-making for this challenging patient cohort.
